# Medicinal Appliances on Sweedish Railroads

**Published:** 1869-08

**Authors:** 


Medicinal Appliances on Sweedish Railroads.—In Sweeden every railroad train has a complete pharmacy and a competent medical staff, so that in case of accident the wants of the wounded are immediately attended to.

